# Public engagement with air quality data: using health behaviour change theory to support exposure-minimising behaviours

**DOI:** 10.1038/s41370-022-00449-2

**Published:** 2022-06-28

**Authors:** Amy McCarron, Sean Semple, Christine F. Braban, Vivien Swanson, Colin Gillespie, Heather D. Price

**Affiliations:** 1 Biological and Environmental Sciences, University of Stirling, Stirling, UK; 2 Institute of Social Marketing and Health, University of Stirling, Stirling, UK; 3 UK Centre for Ecology and Hydrology (UKCEH), Penicuik, UK; 4 Psychology, University of Stirling, Stirling, UK; 5 Scottish Environment Protection Agency (SEPA), Stirling, UK

**Keywords:** Air pollution, Personal exposure, Health studies; Behaviour change

## Abstract

Exposure to air pollution prematurely kills 7 million people globally every year. Policy measures designed to reduce emissions of pollutants, improve ambient air and consequently reduce health impacts, can be effective, but are generally slow to generate change. Individual actions can therefore supplement policy measures and more immediately reduce people’s exposure to air pollution. Air quality indices (AQI) are used globally (though not universally) to translate complex air quality data into a single unitless metric, which can be paired with advice to encourage behaviour change. Here we explore, with reference to health behaviour theories, why these are frequently insufficient to instigate individual change. We examine the health behaviour theoretical steps linking air quality data with reduced air pollution exposure and (consequently) improved public health, arguing that a combination of more ‘personalised’ air quality data and greater public engagement with these data will together better support individual action. Based on this, we present a novel framework, which, when used to shape air quality interventions, has the potential to yield more effective and sustainable interventions to reduce individual exposures and thus reduce the global public health burden of air pollution.

## Introduction

Air pollution is the world’s greatest single environmental health threat, resulting in an estimated 7 million premature deaths globally every year [1]. The health effects associated with exposure to air pollution include acute health impacts such as asthma attacks, and more chronic illnesses such as stroke, chronic obstructive pulmonary disease and lung cancer [2]. Sources of air pollution are numerous and include industry, transport, households, other human activities and natural sources [3]. Acknowledging the importance of good air quality for health, the environment, society and the economy, the United Nations has incorporated improving air quality into its Sustainable Development Goals, namely within SDG 3 (Health and Wellbeing), SDG 7 (Affordable and Clean Energy) and SDG 11 (Sustainable Cities and Societies) [4].

Public policy is a key strategy for improving air quality and people’s air pollution-related health. For example, the 1979 UNECE Convention on Long-Range Transboundary Air Pollution has reduced emissions of harmful pollutants by between 40 and 80% and prevented 600,000 premature deaths every year since 1990 in Europe and North America [5]. Similarly, the State Council of China’s Air Pollution Prevention and Control Action Plan introduced in 2013, successfully reduced annual average concentrations of PM_2.5_, SO_2_ and CO by 33%, 54% and 28%, respectively, resulting in an estimated 47,000 fewer deaths by 2017 [6]. However, public policy as an air quality improvement strategy can be problematic; most policies designed to reduce air pollution focus on outdoor spaces rather than indoor environments where people spend most of their time [7] and public policies are more often very slow to take effect [8]. For example, in 2017 the UK government announced the ban of the sale of diesel and petrol cars in the UK by 2040, over 20 years after its conceptualisation [9], and only recently (in 2020) have brought this forward to 2030. While public policy remains a key strategy for reducing air pollution, individual actions can play a vital and complementary role in placing the individual in control to reduce their exposure to air pollution [10].

Air quality policy is assessed and evaluated based on data from traditional static monitoring stations which undergo rigorous calibration and maintenance to ensure the output data are highly accurate, precise and comparable [11]. However, the outdoor static monitoring network does not represent individuals’ exposure and is not designed to provide information about indoor exposures or to support the ‘personalisation’ of air quality data (i.e. enabling individuals to measure their own exposures based on their individual behaviour and time-activity patterns). Recent advances in sensor technology have resulted in lower cost sensors of variable quality [12] supplementing the static infrastructure in many contexts and, in some cases, is the only viable monitoring option owing to economic, infrastructural, or political factors [13, 14]. While these sensors may be used to monitor air quality in outdoor or indoor contexts, many can also be used to monitor personal exposure by an individual wearing a sensor [15, 16]. A person’s exposure to air pollution will be unique to them and depends on numerous factors including their geographic location, time-activity patterns, occupation, gender and socio-economic status [17]. Such personalisation of air quality data may support the design and implementation of individual plans to reduce exposures (e.g.[18]).

Consulting air pollution levels (using data from static or personal monitors) is one of the recommended individual action strategies for improving air pollution-related health risk [19]. This, however, requires that air quality data are available and accessible, and, furthermore, for this to inform individual behaviour change, individuals must be able to interpret the information provided. Moreover, exposure to air pollution is an environmentally and societally complex and ‘wicked’ problem [20], with various sources producing a ‘cocktail’ of air pollution and therefore no singular ‘correct’ approach or definitive action strategy to reduce exposures. Transcending environmental science, health psychology and public health, tackling air pollution exposure requires transdisciplinary, collaborative and innovative approaches towards a common goal. A fundamental part of this is the inclusion of multiple stakeholders, such as governments, institutions, academia and civil society [21], with the participation of civil society in particular crucial to the formulation of multiple solutions and action strategies that are acceptable and feasible to the general public [21].

The aim of this paper is to explore the theoretical steps linking air quality data to behaviour changes that improve people’s air pollution-related health. Through an evaluation of different types of air quality data and methods to engage people with such data to promote behaviour change, we argue that a combination of ‘personalisation’ of air quality data and enhanced public engagement with these data will support individual action to reduce exposure to air pollutants and consequently improve public health.

### The theoretical basis for generating behaviour changes from air quality data

**Fig. 1:**
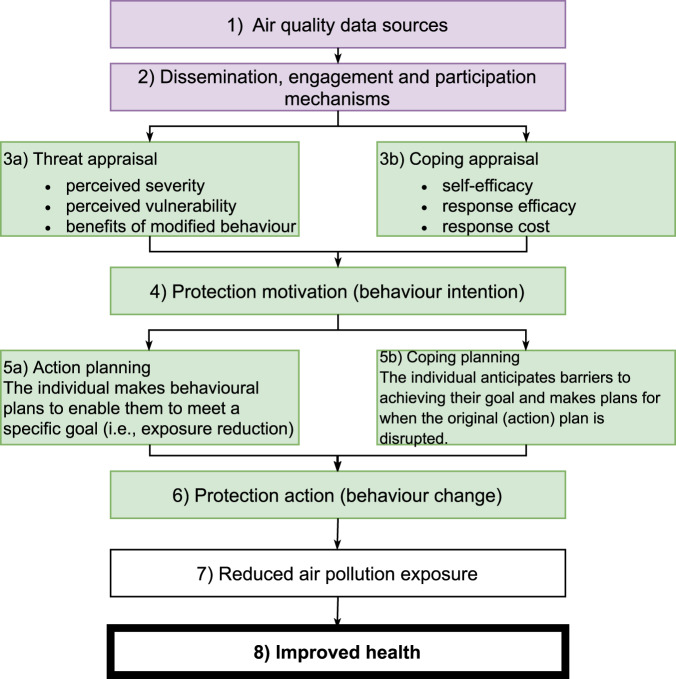
A multistage process to improve air pollution-related health. For air quality data to influence exposure reduction for improved public health requires a multistage process comprising of external (purple) and internal (green) factors. Internal factors are integrated into the process through (adapted) Protection Motivation Theory (PMT; boxes 3a, 3b and 4) and a section of the Health Action Process Approach (HAPA; boxes 5a, 5b and 6).

Accessing air quality data does not automatically induce changes in behaviour that reduce air pollution exposure and improve public health [22]. Rather, it is a first step in a multistage process comprised of external and internal cues motivating and facilitating individual behaviour change which can potentially, in turn, improve public health (Fig. 1).

Air quality data (Fig. 1; Box 1) can be generated from a variety of sources ranging from passive samplers and low-cost sensors to in-situ continuous ambient air quality monitoring stations and remote-sensing [23]. The data arising from these sources can provide various types of information about air quality including focusing on different pollutants and providing data at different spatiotemporal scales (from individual to global and from every second to annual). From this high-level perspective, there is no immediate expectation that raw air quality data from any source will encourage individual behaviour change, however, the quantification of air pollution (which can be an imperceivable and oftentimes invisible problem) is an important starting point. For data to induce behaviour change, regardless of data source, the public need to be able to access, interpret and be motivated to use these data.

For data to be accessed and used by the public, air quality dissemination strategies and engagement tools are needed (Fig. 1; Box 2). This stage, as the ‘public-facing’ part of air quality data and information, is the critical bridge between external raw data (Fig. 1; Box 1) and internal cues to generate individual motivation to reduce exposure (Fig. 1; Box 3a and 3b) and can be considered as a spectrum of approaches. This spectrum of approaches fits well with Jules Pretty’s [24] ‘typology of participation’, most prominently corresponding to *passive*, *consultative*, *functional* and *interactive* participation. *Passive* participation is typified by top-down unilateral announcements used to inform the public and raise awareness. *Consultative* participation approaches are characterised by ‘traditional’ methods including focus group discussions, interviews and questionnaires which have been designed to investigate predetermined aims and predefined problems. Beyond this, *functional* participation, tends to be more interactive and involve citizens to meet predefined objectives. Finally, *interactive* participation involves interdisciplinary methodologies seeking multiple perspectives with citizens participating in joint analysis and the development of action plans, taking control over local and individual decisions. Co-production projects, bringing together academics and non-academics [25] to tackle transdisciplinary, ‘wicked’ issues sits within *interactive* participation (e.g.[26]).

As external cues, data and dissemination, engagement and participation approaches can motivate health protection motivation (Fig. 1; Box 4). However, the extent to which this happens is ultimately shaped by an individual’s assessment of the potential of the threat and their own control over adaptive responses to the threat, as intermediary steps and internal cues (Fig. 1; Box 3a and 3b). Health self-protection motivation (which preludes behaviour change according to Rogers’ [27] Protection Motivation Theory (PMT)), stems from an individual’s threat and coping appraisal. Threat appraisal (Fig. 1; Box 3a) consists of the assessment by an individual of the perceived severity of the threat (degree of harm), their vulnerability to the threat (likelihood of experiencing harm) and the benefit of behaviour modification. Coping appraisal (Fig. 1; Box 3b), rather than assessing the threat itself, is a process which assesses the response efficacy (the effectiveness of the adaptation of behaviour), the response cost (the cost of performing the behaviour change i.e. financial, time, convenience) to cope with and avoid the threat, and the individual’s self-efficacy (the belief that they can successfully conduct the change in behaviour). Therefore, altering behaviours by applying PMT can be about altering perceived self-efficacy and perceived control as well as giving individuals’ *actual* behavioural control. For effective interventions, first the risk needs to be conveyed (i.e. in the communication of air quality information), before then presenting the preferred behaviour as a simple, effective and low-cost solution to minimise the risk [28].

Whilst these factors are important motivators for behaviour change, a further step is required for translation into protection action (Fig. 1; Box 6) to bridge the motivation-behaviour gap [29]. Where PMT explains the role of risk perception as one aspect of motivation, Schwarzer’s [30] Health Action Process Approach (HAPA) explains that action and coping planning are prerequisites of actual (rather than intentional) behaviour change, with the enactment of behaviours included within HAPA and helps to bridge the motivation-behaviour gap whereby planning is a key stage between motivation and behaviour. Action plans (Fig. 1; Box 5a) are formed by the individual to decide in what situation they will perform a specific behaviour to meet a specific goal (e.g. “To reduce my exposure to air pollution, I will avoid walking along busy roads on my commute to work”). Coping plans (Fig. 1; Box 5b) connect coping responses to situations that can jeopardise one’s goal achievement (e.g. “If I am leaving for work and air quality is poor, then I will wear a facemask while walking”). Ultimately these planning processes place the individual at the core of the behaviour change, allowing an individual assessment of feasibility and acceptability. The individual is placed at the centre- or the core- of Social Ecological Models (e.g.[31], which recognises that individual’s behaviours (and behavioural determinants) vary and are shaped by multilevel influences, not only at the individual level (e.g. personal beliefs), but by social (e.g. norms) and environmental (e.g. situational) factors also. To the authors’ knowledge, the socio-ecological model has not been directly applied in an air quality-specific context. In the exposure minimisation context, shifting from motorised to active transport, moderating outdoor physical activities in poor air conditions, using ‘cleaner’ household fuels and staying indoors during pollution episodes [19, 32] are examples of protection actions (Fig. 1; Box 6). Protection actions must be considerate of the individual context. The COM-B model [33] also focuses on the person in context, and notes that for any behaviour change intervention to be effective, three factors need to be present at the individual level: capability, opportunity and motivation. Only when an individual has the capability and opportunity to engage in the preferred behaviour (determined by physical and psychological capability, and physical and social environments for opportunity), and is motivated to enact the preferred behaviour over any other behaviours, will a behaviour change occur [33]. The COM-B model has been used to promote behaviour change in air quality-related interventions. For example, D’Antoni et al. [34] used the components of COM-B to design smartphone air quality alerts, finding the theory-based intervention more successfully made participants consider more permanent behaviour changes to reduce exposures. Similarly, Thompson et al. [35] used COM-B to inform a cookstove intervention. The COM-B model has also been used as part of an indoor air quality intervention evaluation, successfully highlighting the components which act as barriers to behaviour change in relation to indoor air quality (e.g.[36]. Health and behaviour change theories aid our understanding of the mechanisms of action and thus can lead to more effective interventions to improve health behaviours. Taking a theory-driven approach to air quality data generation and communication is needed to reduce air pollution exposure (Fig. 1; Box 7) and improve public health (Fig. 1; Box 8).

Air quality data (Fig. 1; Box 1), and dissemination, engagement and participation approaches (Fig. 1; Box 2), are external malleable cues that feed directly into threat and coping appraisal, making them key stages to target in order to inform and influence an individual’s protection motivation. For subsequent protection action to occur, the personal context is key. These are critical first steps as part of a multistage process to reduce individuals’ exposures and improve public health.

In the following section we outline the traditional mechanisms used to promote individual behaviour change to reduce air pollution exposure, examining its underpinning data (section *AQI data sources*) and the mechanisms by which air quality information is shared (section *AQI dissemination mechanisms*) separately.

### Traditional mechanisms to promote individual exposure minimising behaviours

**Fig. 2:**
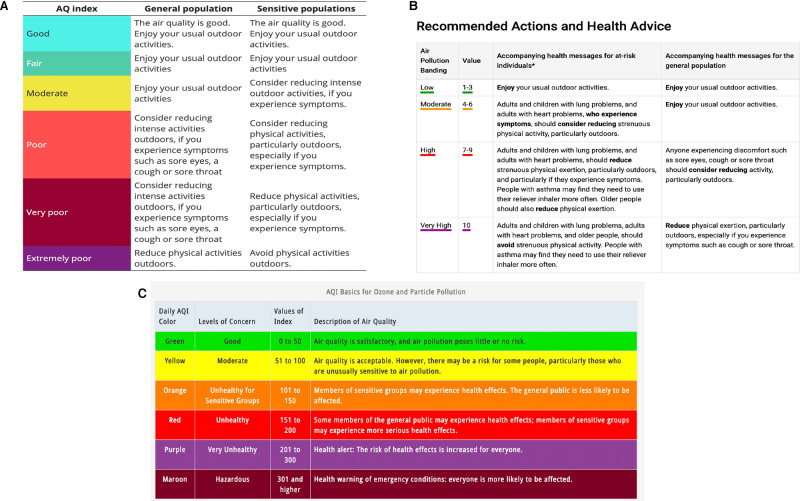
Air Quality Indices. Examples of the information available as part of various AQI including the EU CAQI (**A**), UK DAQI (**B**) and US AQI (**C**).

The traditional suite of mechanisms used to promote individual behaviour change are ‘top-down’ in terms of both data sources and citizen involvement, whereby active data *dissemination* and public *informing* are frequent. A key example of this is the Air Quality Index (AQI) which is a common tool employed in communicating air pollution information to the general public [37]. Different AQI are used globally (e.g. EU Common Air Quality Index [CAQI], UK Daily Air Quality Index [DAQI], US AirNow AQI; Fig. 2A, B, C, respectively) to describe air pollution as an understandable standardised summary of ambient air quality and its associated health risk to the public [38]. Where AQI are available, data are frequently converted from physical units to a unitless index value, which, though it may compromise precision and accuracy [32], is an effort to increase public accessibility and understanding of air quality data [39]. Here we examine the underpinning data (section *AQI data sources*) and the dissemination strategy of the AQI (section *AQI dissemination mechanisms*), exploring its potential for generating individual behaviour change.

### AQI data sources

AQI calculations are most often based on data from static regulatory monitors and many monitoring networks are structured around a country’s commitment to report air quality data and modelled forecasts to the general public [40]. Data collected from static site monitoring stations are generally accepted to be representative of average ambient concentrations within the local community [41], and provide highly accurate and precise monitoring data [42]. Though most frequently the raw data from regulatory monitoring stations are accessed and used by researchers, governments and industry via government websites and research databases [42], the output data from these monitors are available to the wider public in only some countries in a ‘fully-open’ manner [43]. Air quality data that are actively shared with the public (see section *AQI dissemination mechanisms*) tend to be converted to an AQI. Though it has been argued that variances between AQI at country-level makes comparisons of values challenging [44–46] and most ‘daily’ AQI fail to report short-term peaks, these are often the highest spatiotemporally resolved data available to the general public.

The ability of the data communicated as part of the AQI to motivate behaviour change (i.e. protection motivation; PMT) assumes that the public understand and engage with the AQI and accessing the AQI promotes self-protection behaviours (i.e. protection action; HAPA) [18]. However, evidence for the AQI in engaging and enabling exposure reduction behaviours is limited [19, 22]. D’Antoni et al[47]. found that, despite AQI alerts increasing perceived severity (magnitude of negative health consequences of exposure to air pollutants), the perceived susceptibility (personal vulnerability) was a barrier to behaviour change. This suggests that though the AQI can successfully communicate the risk of air pollution exposure, these remain as distant problems with impersonal risks [48], thereby unable to influence or demonstrate perceived vulnerability in one’s own threat appraisal (Fig. 1; Box 3a). In addition, it must also be considered that AQI may have an unintended effect on threat appraisal, particularly in settings with generally ‘good’ (according to the AQI) ambient air quality. The unintended interpretation of an AQI value suggestive of ‘good’ air quality will shape risk perception [49] and can diminish the sense of threat posed by air pollution more generally, despite there being no safe threshold level of exposure below which no adverse health impacts occur [2]. Personalising AQIs and air quality data (e.g. by characterising air pollution in the more immediate local or home environment) has the ability to personalise risk, influence threat appraisal and help promote individual protection motivation. For example, communicating personal vulnerability via personalised air quality data has been found to help individuals revisit their prior perceptions about air pollution and demonstrate the impact individual actions have on personal exposures [50]. In another study, providing participants with personal sensors on the commute to school resulted in the majority identifying air pollution as a ‘threat’, caused many to perceive air pollution as a greater ‘problem’ on the school commute than previously thought and resulted in the majority of participants taking protective action in response to the monitoring data [51].

Increasing the representativeness of air quality data, in addition to its potential to alter threat appraisal, has potential to alter one’s coping appraisal (Fig. 1; Box 3b). Lack of self-efficacy has been identified as a barrier to adherence to AQI-recommended behaviours [47]. Perceived behavioural control, as a distinct but related construct to self-efficacy, is one of the most important psychological factors for behaviour change [52]. As a dynamic but vital determinant of behaviour, perceived behavioural control (in addition to actual behaviour control) needs to present in coping appraisal for protection motivation. Particularly regarding air pollution (ambient especially), which is sometimes considered as a ‘distant’ and uncontrollable problem, creating perceived behavioural control is fundamental to behavioural intentions and change. Bandura [53] identified that individuals’ efficacy beliefs are based upon four factors: mastery experiences, vicarious experiences, verbal persuasion and physiological states. Mastery experiences (or performance outcomes) are the experiences gained by altering behaviour successfully and the most influential source is the interpreted result of an individual’s previous performance [53]. More personally representative data can demonstrate behaviour change performance outcomes (i.e. response efficacy) and simultaneously increase self-efficacy. For example, Wong-Parodi et al. [54] found that microenvironmental air quality data can help people make the connection between exposure, attitudes and behaviour change actions, and found that subsequent to sensor use, participants felt more confident about knowing how to mitigate the risk of exposure, as well as participants tending to take more action to reduce pollution. Similarly, Bales et al. [55] noted that participants were more “empowered” to alter their behaviours and reported individual changes such as avoiding busier roads when walking, reducing idling, and avoiding bus exhaust fumes. In these instances, more personally representative data have increased self-efficacy (and perceived behaviour control as a related construct), response efficacy and demonstrate benefits to change, thus protection motivation. Together, this suggests that making air quality data ‘more personal’ has the potential to encourage behaviour changes that reduce exposure and improve air pollution-related health.

### AQI dissemination mechanisms

The AQI is designed for the active dissemination of air quality information to the public for the protection of public health [56]. As such, the AQI has traditionally been disseminated via television, radio and newspaper [57], forecasting aggregate pollution levels and offering (primarily avoidance) behaviour advice. As technology and how we use it has advanced, so too have the various dissemination strategies. Now AQIs are, where available, frequently accessible via government, environment agency and third-sector websites and apps- both specific (e.g. IQAir AirVisual) and non-specific (e.g. weather and maps apps) to air quality- and increasingly on social media. These are *passive* participation methods according to Jules Pretty’s ‘typology of participation’ [24], characterised by unilateral announcements without citizen input with unbalanced power dynamics between the lay public and researchers/officials. This resembles a one-way flow of information from officials to the public, which has key advantages around efficiency, cost-effectiveness and awareness raising [58].

However, this dissemination approach relies on the public understanding and interpreting AQIs, which has been previously identified as a barrier to behaviour change [47, 59, 60]. Reflecting on the complexities of air quality information and difficulties interpreting this by the public, Hubbell et al. [61] recommend two-way dialogue between air quality monitoring experts and the lay public, and it has been suggested that engagement with the general public is required (over simply providing data), to support individual behaviour change [62]. Ultimately, informing people about high pollution episodes via traditional dissemination strategies such as AQI alerts or advisories has had limited effectiveness [63] and though information provision has importance, it is insufficient, on its own, to trigger behaviour response [22].

Public engagement is believed to be a key part of the solution when it comes to exposure minimising behaviour change (e.g.[60, 64, 65]). For interventions to promote behaviour change, ‘one size fits all’ does not work [66, 67]. Information is effective for behaviour change, not due to its accuracy, precision or completeness, but the extent to which it captures its audience, gains their involvement and overcomes scepticism [68]. Issue involvement is a key moderator in shaping an individual’s attitude or favourability towards a message [69] and thus its ability to persuade for behaviour change (i.e. adherence to the suggested behaviour of the AQI). Messages with high issue involvement have a high degree of personal relevance to the recipient [69], and in turn are more likely to induce attitude change via central route processing (that is, the individual carefully considers, elaborates and engages with the persuasive message [see Petty and Cacioppo [70] *Elaboration Likelihood Model*]) since the issue is of direct interest to them. Attitude change via central route processing is more likely to be sustained and stable [28]. It has been argued that the health and behaviour messages communicated as part of AQI advisories do not effectively support individual behaviour change [59], owing to their lack of specificity [71]. Applying the theory of issue involvement and the *Elaboration Likelihood Model*, AQI could be more persuasive for behaviour change if more engaging and using more personally involving, specific and tailored messages (and data, see section *AQI data sources*).

### An expanded approach for public engagement with air quality data

Using the key example of the AQI, we argue that traditional approaches to supporting behaviour change through the dissemination of air quality data have limited effectiveness. Following on from this, we propose that by; (1) supplying people with more personally representative data (or supporting people to collect their own data) (section *More personally-representative data*); and (2) engaging people in the process (section *More participatory engagement*), we can better support individuals to change their behaviours and improve their air pollution-related health. We discuss these ideas in turn below, before considering the benefits of combining these two approaches in section *Pairing personally relevant data with participatory engagement*.

### More personally-representative data

Rapid advances in sensor technology are revolutionising air pollution monitoring [42]. Instead of having few or no static air quality measurement stations to characterise the air quality of a geographic area, the development of smaller, cheaper, portable sensors has enabled air pollution measurements by various users and for a wider variety of purposes [72]. These sensors have commonly been used to investigate air pollution concentrations in specific microenvironments (e.g.[73]), in exposure assessment studies (e.g.[15]) and in behaviour change intervention studies (e.g.[74]), and their use in ambient air pollution monitoring studies is also growing [75]. There are numerous critiques of these sensors for measuring air pollution, particularly around accuracy [76], robustness, repeatability [11], reliability [77], nominal range and response time [78] compared to reference-grade monitors. These limitations must be communicated openly and clearly to the public to ensure appropriate data interpretation and risk perception. However, these limitations are balanced against the relatively cheaper cost of sensors, the ability of the sensors to demonstrate relative change in exposure, the ability to get more people involved in measurements and the potential increase in spatiotemporal resolution of generated data (e.g.[79]), in addition to the benefit of allowing for monitoring where otherwise regulatory monitoring is not economically or politically viable [13, 80].

Though increasing the representativeness of data has the potential to alter threat and coping appraisal for protection motivation (see section *AQI data sources*), this has not been found universally. For example, both Boso et al. [81] and Oltra et al. [18] found that having access to a sensor (compared with only having access to ‘traditional information’ analogous to that provided in advisories or AQI) generated increased awareness among participants, however a low sense of self-efficacy and control over personal exposure remained. Similarly, Varaden [82], in a participatory monitoring study conducted with school children, found that awareness of air pollution was raised among most participants after taking part in the study and increased parents’ sense of autonomy over their children’s exposures, while positive protection action was reported in a much smaller proportion of participants. Lastly, despite Heydon and Chakraborty [51] finding that sensors increased awareness, threat appraisal and changed participants’ behaviours, they found that when participants were unable to reduce the risk (evidenced by exposure data during a follow-up monitoring campaign), this led to inaction. Together, these examples demonstrate the complexity and nuance associated with behaviour change in relation to the ‘wicked’ problem of air pollution and suggests that greater support is required to transform air pollution awareness into protection action. Oltra et al. [18] acknowledged that behavioural interventions need to take internal and external determinants into account, and simply increasing information availability does not always prompt individual action [83]. Therefore, while increasing data representativeness is a fundamental component to better support exposure minimising behaviours, alone it is not enough to guarantee the generation of protection action.

### More participatory engagement

Public engagement can span a spectrum of approaches designed to generate two-way dialogue with the public. Engagement approaches can range from more ‘traditional’ and *consultative* mechanisms, such as focus group discussions, interviews, and questionnaires, to more *interactive* and creative engagement methods. Traditional engagement mechanisms may generate some dialogue to better understand community perceptions of air pollution (e.g.[84]) and drivers of behaviour change. However, their ability to generate protection action through participation are constrained by the frequently limited depth and/ or scope and the focused research agenda of studies undertaken [58, 85]. Creative methods, ranging from physical events, such as street art and creative play, to more technologically driven, including for example drone imagery and wearable cameras [86], are *interactive* by design and can support two-way dialogue between researchers and participants [86] and generate participant relevance, uncover lived experiences, build individual confidence and capacity, facilitate solution-orientated discourse and stimulate actions [86, 87]. Focussing on storytelling and theatre as specific examples of creative *interactive* participatory methods, we examine their efficacy for generating protection motivation and protection action.

Storytelling, as a tool for learning, empathising, educating, reflecting and advocating [88], has the potential to influence change in attitudes, behaviour, culture and policy [89]. Behaviour change is not generated from scientific knowledge, but from dialogues created between a listener and teller, and more personalised communication offers the opportunity for social change [90]. Storytelling places more emphasis on actions and consequences with more exploration of emotional, psychological and cultural matters [91] drawing on past knowledge and experiences, and making it relevant with the present [92]. This can be used to engage communities and give a voice to those usually without, in a manner very different from traditional scientific or governmental communications, and allows individuals to express complex thoughts and feelings through a narrative relatable and understandable by themselves [93]. Knowledge that is incorporated into storytelling, in a manner different than traditional scientific communications, generates greater engagement, attention [94] and willingness to act [95, 96]. While Dahlstrom [94] described storytelling as a tool to communicate with nonexpert audiences, it can be argued that in fact it is a tool to enable narrative between different types of expert. Stories can draw on memories and emotions and stimulate actions [86] that data and statistics simply cannot.

Theatre for Development, developed by Boal for the ‘oppressed’, works across individual, group and social levels, using visual and tangible interaction to disrupt language, literacy and educational barriers that may otherwise limit engagement and fail to explore the full extent of feasible solutions, whilst simultaneously promoting tools for behaviour change [97]. Theatre for Development is an umbrella term used to describe many different types of theatre including forum, legislative, image and invisible theatre, which range in how they fit within the ‘typology of participation’ and the extent to which they include the lay public as active participants. Theatre for Development (of any kind) is proactive, not only acknowledging the existence of a problem, but actively seeking feasible solutions to said problem. Focussing specifically on forum theatre, whereby the audience is comprised of community members who share similar lived experiences, forum theatre is a form of *interactive* participation and an audio-visual tool in which participants (known as spect-actors [98]) spectate as an audience and can interact and participate as an actor, joining the scene to change the outcome scenario and help resolve an issue by offering their own solution. This can give those usually unheard, a voice to identify before unconsidered solutions [87], by exploring past and present situations to find solutions as a “rehearsal for the future” ([99], pg. 12). The difficulties of behaviour change in the ‘real-world’ are imitated with the other actors opposing the proposed changes of the spect-actor. West et al. [100], developed forum theatre storylines from community members own accounts of how air quality had affected them and presented the play at various community hubs around Mukuru (an informal settlement in Kenya), allowing community members to contribute to the scene and offer their suggestions for resolving the various issues. It has been suggested that the personal relevance of forum theatre is a key motivator for individual behaviour change [101].

*Interactive* participatory research methods, including creative methods, can result in more effective and sustainable outcomes and solutions [102] and offer an important role in bringing together multiple stakeholders and challenging traditional power dynamics to tackle complex issues [86]. Complex and ‘wicked’ problems, such as air pollution [20], require practical and relevant knowledge which is not best uncovered through traditional research methods and instead requires transdisciplinary, collaborative and innovative approaches. Co-production speaks to participatory research in that it challenges the traditional power dynamics within research, but goes beyond *consultation* or collaboration, and instead is a commitment to working in equal partnership throughout the entirety of the project, with benefits to all parties. In doing so, co-production gives equity to all forms of knowledge, realising that all stakeholders have knowledge and skills of equal importance, and recognises that those affected by a research project are best-placed to design and deliver it [103]. Instead of developing interventions ‘for’, this approach develops interventions ‘with’ relevant stakeholders that fit the needs and priorities of those they impact [104]. For example, West et al. [100], using creative methods in a co-production approach, found that this enabled the production of several solutions to air pollution which were designed around and suitable for the local context informed by communities’ priorities and contributing towards “improved outcomes and achievable solutions” [105]. Where there is a need to induce behaviour change, co-production is believed to be particularly valuable [106], yet the value of such research is only now being realised for the ‘wicked’ issue of air pollution.

### Pairing personally relevant data with participatory engagement

For protection action (or behaviour change) towards exposure minimising behaviours, targeting only the components of protection motivation (i.e. threat and coping appraisal) is insufficient. As this and other papers have identified (e.g.[107]), data representativeness and increased engagement independently have an important role to play in effectively communicating air quality information to the public to generate behaviour change. As distinct features, we discuss their ability to shape and alter threat and coping appraisal (sections *More personally-representative data* and *More participatory engagement*), however as detached and distinct steps, we argue that these are limited in their ability to bridge the motivation-action gap. This paper establishes the value of concurrently targeting data and engagement to evoke behaviour changes that improve people’s air pollution-related health.

Pairing more personally representative data with suitable, enhanced participatory mechanisms has the potential to better support individual behaviour change. This has already been evidenced in the second-hand smoke literature, which, while we acknowledge is a different behaviour to change and is underpinned by different psychological, physiological, social and environmental determinants, does provide a useful comparison. Coupling personalised, low-cost air quality data feedback with motivational interviews (creating two-way dialogue between researchers/healthcare practitioners and participants to increase self-efficacy and create plans, for example), has been found to successfully promote smoking behaviour change [108, 109]. Yet mixed-methods interventions have not been limited to smoking behaviours. Barnes et al. [110] used personalised baseline data as the basis for discussions with participants about their behaviours and possible modifications they could make to reduce household air pollution concentrations. Using a community counselling strategy, starting with knowledge sharing before engaging in discussion over feasible and acceptable behaviour modifications, household PM_10_ and CO concentrations were reduced [110].

**Fig. 3:**
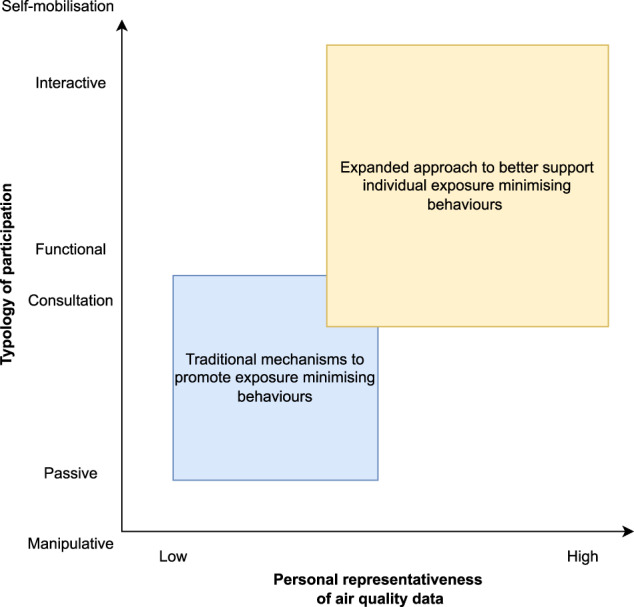
A framework to better support behaviour change. The expanded approach to promote individual behaviour change relies simultaneously on more personally representative data and increased citizen participation moving away from passive participatory processes towards interactive participation (Pretty, 1995). Participation for material incentives has been omitted from the *y*-axis because it does not generally fit within participatory methods used for behaviour change. Self-mobilisation goes beyond engagement towards empowerment and so is outwith the scope of participatory mechanisms.

Building on this previous work, we propose a framework to better support individual behaviour change to reduce exposure to air pollution and improve health (Fig. 3). This framework recognises the importance of air quality data that are more personally representative (*x*-axis) and enhanced participation and engagement of the individuals whose behaviour we aim to change (*y*-axis) as two distinct but coactive variables.

**Fig. 4:**
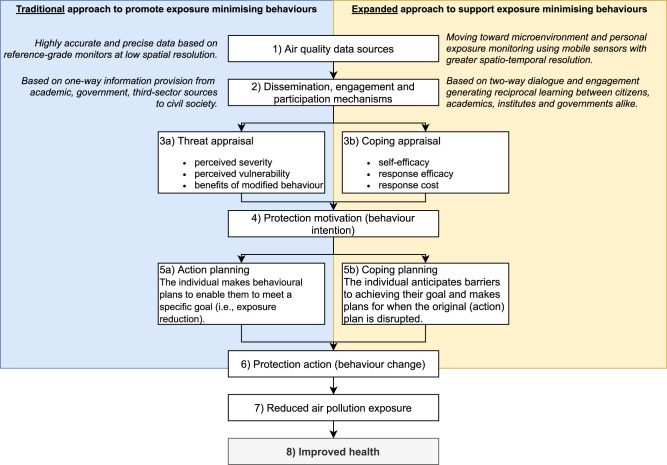
Comparing the ‘traditional’ and ‘expanded’ approach to ultimately improve air pollution-related health. *Left*: the traditional approach to promoting exposure minimising behaviours is based on top-down dissemination using highly accurate data with limited personal representativeness. *Right*: an alternative (‘expanded approach’) approach to supporting exposure minimising behaviours could be more inclusive and collaborative with dialogue between all stakeholders and making use of more interactive data collection methods increasing personal representativeness. Note, both types of appraisal and planning exist in either side of the figure.

A technocentric approach to supporting behaviour change, relying on sensor technology and personal exposure data can only encourage individual exposure minimising behaviours so far (i.e. horizonal trajectory). Similarly, whilst increasing and enhancing citizen participation is positive, only investing resource into this (i.e. vertical trajectory) or capping this at *consultative* or *functional* participation, will not fully support individuals to change their behaviour. To better support individual behaviour change that will reduce air pollution exposure, requires a shift in the diagonal trajectory, adopting tools to both increase data representativeness (Fig. 4; Box 1) and citizen participation (Fig. 4; Box 2) in tandem. Summarising the contrasts in air quality data sources and dissemination, engagement and participation mechanisms between the traditional and expanded approaches (Fig. 4), highlights how, by specifically targeting these key stages (as external variable factors), we have the potential to provoke internal triggers which can spread throughout the multistage process for exposure reduction and to better support the likelihood of achieving improved public health.

In comparison to the traditional approach, the expanded approach does have some key drawbacks, including the resources needed (e.g. human, social and financial), the availability of personalised air quality data, requiring practitioners to have a robust knowledge base, and greater input from citizens (Table 1). A specific concern is the transfer of the weight of responsibility for air quality and air quality-related health away from governments and institutions towards citizens. For this reason, we do not advocate replacing the traditional approach (i.e. regulatory monitoring by governments and institutions and the use of AQIs) with the expanded approach. By adopting the expanded approach, we can gain from the combined benefits of increasing data availability and engaged dialogue between stakeholders to aid the collection, analysis and interpretation of air quality information in a way meaningful to the public. This, in turn, will generate greater citizen autonomy and empowerment over personal exposures. Adopting the expanded approach and using a suite of approaches across the participation- representativeness space (Fig. 3), will better support behaviour changes in relation to air pollution exposures.

**Table 1:** Advantages and disadvantages of the transition from the ‘traditional’ to an ‘expanded’ approach.

	Advantages	Disadvantages
Traditional approach	Though monitoring is expensive to set up and maintain, this is balanced by minimal costs associated with disseminating air quality information which can be automated.	Mismatch between the spatial resolution of the air quality data (community-level) and the actions being encouraged to minimise exposure (individual-level).
	Meets regulatory/legislative need for monitoring and dissemination.	Mechanisms not accessed (or indeed known about) by a large proportion of the population.
	Citizen engagement doesn’t require continual input from researchers/ healthcare practitioners and thus more viable in the longer-term.	In settings without the infrastructural or technical capabilities such mechanisms to promote behaviour change are not yet feasible.
Expanded approach	Personally relevant and tailored solutions taking into account personal capabilities and opportunities.	More resource intensive (i.e. human capital), yet by their nature only work when engaging smaller groups of people.
	Empowers citizens by equipping them with relevant knowledge to gain control over their personal exposures.	Requires researchers/practitioners to have a robust knowledge base, requiring an understanding of air quality, behaviour change psychology and public health.
	Greater opportunity to improve individual and public health.	Continual human input required to facilitate pairing citizen engagement with personally/locally representative data meaning that most work is time limited.
	Rooted within the community meaning greater ownership and trust.	Requires engagement and effort from citizens, weighed against their other more immediate needs and priorities, for it to be successful.

### Recommendations for future work

The expanded approach should be seen as a ‘must do’ rather than a ‘nice to do’ to help combat the health impacts of air pollution. While the theoretical basis for the expanded framework is robust, future exposure reduction studies should evaluate the efficacy of the approach. Many data feedback intervention studies conducted to date- and included within this paper- lack robust evaluation reporting self-reported behaviour change or conducted with a homogenous population (e.g. school children, geographic area). A particular shortcoming within behaviour change studies is the sustainability of the intervention. Longitudinal studies which make a quantitative and qualitative assessment of the sustainability of behaviour change are needed. To this end, more work is needed to understand whether behavioural changes made using the approaches proposed under the expanded approach are sustainable in the longer term. We recommend that further work is undertaken in a variety of global contexts with different population subgroups (e.g. age, education level, pre-existing disease) to further test the potential for a combination of personalised air quality data and enhanced engagement to lead to reduced air pollution exposure and improved health. In particular, there is a need to explore the potential of co-production approaches, where participants are involved in all stages of the research process to support behavioural changes. Keeping in mind the individual level differences in engaging in protective behaviours, future work should have emphasis on exploring individuals’ capabilities, opportunities and motivations for behaviour change with respect to air pollution exposure protective behaviours and should consider the Social Ecological Model, starting with the individual at the core. Generating autonomy and prompting protection action requires working not only across disciplines, but also across stakeholder groups, and placing greater emphasis on the co-production of air quality projects that involve civil society, researchers and policymakers equally in the conception through to analysis and dissemination stages of projects is a key part of this. However, for improved public health this needs to reach beyond personal exposure autonomy; more emphasis is needed on population exposure and the role individual behaviours play in modifying local concentrations of pollutants.

## Conclusion

In this paper we have shown that participation mechanisms and their underpinning air quality data are two distinct but related key external steps preceding health protection motivation, protection action, reduced exposure and improved public health. As external cues which lead directly to (and can influence) internal determinants of behaviour change, these are crucial in shaping an individual’s threat and coping appraisal and are the first steps in a multistage process for improved public health. Considering the traditional approaches to the promotion of exposure minimising behaviours regarding these key stages from a health psychology perspective, it is apparent that they fail to support significant individual protection motivation and protection action. Examining alternative approaches to both data sourcing and citizen participation and evaluating their success at targeting the psychological elements of protection motivation, we argue that both increasing the personal representativeness of air quality data and increasing citizen involvement can better support protection action when used simultaneously.

Top-down, government policy is vital to reduce the health impacts of air pollution but can (and should) be supported by individual action. We acknowledge that the expanded approach represents a resource-intensive approach that will not be achievable in all global locations and that it requires citizens to have high protection motivation, the capacity and interest to be ‘engageable’ with the topic. The expanded approach framework proposed in this paper is also not attempting to promote personal monitoring or participatory methods as ‘silver bullet’ techniques, instead it is an attempt to highlight the additional benefits such methods can have on behaviour change and motivation at the individual level. Additionally, this paper is not proposing a shift away from traditional, static, regulatory monitoring. Simply, from a behaviour change perspective, the evidence presented in this paper suggests that such an approach is not adequate to support personal protective action against air pollution exposure.

Air pollution is a major health and sustainability challenge of modern times. While not a panacea for the ‘wicked’ problem of air pollution, making air quality data more personal and involving citizens in research processes simultaneously has the potential to support the reduction of the global public health burden of air pollution and accelerate progress towards the SDGs.
